# Species-Specific Activity of SIV Nef and HIV-1 Vpu in Overcoming Restriction by Tetherin/BST2

**DOI:** 10.1371/journal.ppat.1000429

**Published:** 2009-05-15

**Authors:** Bin Jia, Ruth Serra-Moreno, William Neidermyer, Andrew Rahmberg, John Mackey, Ismael Ben Fofana, Welkin E. Johnson, Susan Westmoreland, David T. Evans

**Affiliations:** 1 Department of Microbiology and Molecular Genetics, Harvard Medical School, New England Primate Research Center, Southborough, Massachusetts, United States of America; 2 Department of Pathology, Harvard Medical School, New England Primate Research Center, Southborough, Massachusetts, United States of America; Fred Hutchinson Cancer Research Center, United States of America

## Abstract

Tetherin, also known as BST2, CD317 or HM1.24, was recently identified as an interferon-inducible host–cell factor that interferes with the detachment of virus particles from infected cells. HIV-1 overcomes this restriction by expressing an accessory protein, Vpu, which counteracts tetherin. Since lentiviruses of the SIV_smm/mac_/HIV-2 lineage do not have a *vpu* gene, this activity has likely been assumed by other viral gene products. We found that deletion of the SIV_mac_239 *nef* gene significantly impaired virus release in cells expressing rhesus macaque tetherin. Virus release could be restored by expressing Nef in *trans*. However, Nef was unable to facilitate virus release in the presence of human tetherin. Conversely, Vpu enhanced virus release in the presence of human tetherin, but not in the presence of rhesus tetherin. In accordance with the species-specificity of Nef in mediating virus release, SIV Nef downregulated cell-surface expression of rhesus tetherin, but did not downregulate human tetherin. The specificity of SIV Nef for rhesus tetherin mapped to four amino acids in the cytoplasmic domain of the molecule that are missing from human tetherin, whereas the specificity of Vpu for human tetherin mapped to amino acid differences in the transmembrane domain. Nef alleles of SIV_smm_, HIV-2 and HIV-1 were also able to rescue virus release in the presence of both rhesus macaque and sooty mangabey tetherin, but were generally ineffective against human tetherin. Thus, the ability of Nef to antagonize tetherin from these Old World primates appears to be conserved among the primate lentiviruses. These results identify Nef as the viral gene product of SIV that opposes restriction by tetherin in rhesus macaques and sooty mangabeys, and reveal species-specificity in the activities of both Nef and Vpu in overcoming tetherin in their respective hosts.

## Author Summary

Tetherin was recently identified as a host–cell factor that interferes with the detachment of virus particles from infected cells. HIV-1 overcomes the antiviral effects of tetherin by expressing Vpu, which mediates the degradation of tetherin. While tetherin has broad activity against diverse types of viruses, only a few of the primate AIDS viruses express Vpu. Simian immunodeficiency virus (SIV) does not have a *vpu* gene. Since SIV infection of the rhesus macaque is an important animal model for AIDS vaccine development, we set out to determine how SIV overcomes restriction by tetherin in this species. We found that the SIV Nef protein could counteract rhesus macaque tetherin, but not human tetherin. Conversely, the HIV-1 Vpu protein counteracted human tetherin, but not rhesus tetherin. The specificity of Nef for rhesus tetherin mapped to four amino acids in the cytoplasmic domain of the molecule that are missing from human tetherin, whereas the specificity of Vpu for human tetherin mapped to amino acid differences in the transmembrane domain. These observations identify a role for the SIV Nef protein in counteracting tetherin, and reveal species-specificity in the activities of both Nef and Vpu in overcoming tetherin in their respective hosts.

## Introduction

Efforts to elucidate the function of the HIV-1 Vpu protein recently led to the identification of an interferon-inducible, host-cell factor that interferes with the detachment of virions from infected cells [1],[2]. *Vpu*-deleted strains of HIV-1 exhibit a cell-type dependent defect in the release of virus particles from cells [3],[4],[5],[6],[7],[8]. In certain human cell lines, such as HeLa, Hep-2 and Jurkat cells, virus particles assemble and bud from the plasma membrane, but fail to detach from the cell surface [6],[9],[10]. These particles then become internalized where they accumulate in endosomal compartments [6],[9]. In other cell types, such as 293T, HOS and Cos-7 cells, Vpu is not required for virion release [9],[10]. Varthakavi and Spearman demonstrated that heterokaryon fusions of permissive and non-permissive cells exhibited a non-permissive phenotype for the release of *vpu*-deleted HIV-1 [10]. These experiments pointed to the presence of an inhibitor of virus release in *Δvpu*-restrictive cells [10]. Subsequent studies revealed that the putative restriction factor was an interferon-inducible protein exposed on the cell surface [9],[11]. These observations quickly led Neil and Bieniasz to identify bone marrow stromal antigen 2 (BST2), also known as CD317 or HM1.24, from an expression analysis of IFNα-treated cells as the cellular gene product responsible for the restriction of *vpu*-deficient HIV-1 [1]. Based on a proteomic analysis of viral modulators of cell membrane proteins that was actually the first study to suggest a role for Vpu in the downmodulation of BST2 [12], Van Damme and Guatelli independently identified BST2 as the restriction factor for HIV-1 *Δvpu*, and further demonstrated that Vpu-mediated downregulation of BST2 facilitated virion release [2]. For its role in inhibiting the detachment of virus particles from the surface of infected cells, BST2 was re-named “tetherin” [1].

Tetherin is an integral membrane protein with a number of peculiar features that suggests it plays a direct role in inhibiting the release of virions from infected cells. The N-terminus of the molecule is located in the cytoplasm, followed by a transmembrane domain and an extracellular coiled-coil domain [13],[14]. The C-terminus of the protein contains a predicted cleavage site for the addition of a glycosyl-phosphatidylinositol (GPI) anchor [13],[14]. Hence, tetherin is predicted to adopt an unusual topology in which both ends of the protein are anchored in the cell membrane [13],[14]. Tetherin also associates with cholesterol-rich lipid rafts [13], which have been implicated as sites of virus assembly and release for HIV-1 as well as for other enveloped viruses [15],[16],[17],[18],[19]. Thus, it has been proposed that tetherin may become incorporated into enveloped viruses as they attempt to bud from the cell surface and may prevent their release by bridging them to the plasma membrane [1],[2],[20]. Interactions between the cytoplasmic domain of tetherin and the cellular endocytosis machinery may then mediate the internalization of captured virions for degradation within endosomal compartments [1],[2],[9],[11],[20].

Among the primate lentiviruses, only the HIV-1/SIV_cpz_ and SIV_gsn/mon/mus_ lineages are known to have a *vpu* gene [21],[22],[23],[24]. Given evidence that tetherin has broad antiviral activity against a diverse range of enveloped viruses, including retroviruses, filoviruses and arenaviruses [1],[25],[26],[27], it is likely that other lentiviruses have also evolved countermeasures to overcome restriction by tetherin. In the case of HIV-2, the envelope glycoproteins of certain isolates have Vpu-like activity that can enhance the release of virions from restrictive cells [28],[29],[30],[31]. Phylogenetic and epidemiological evidence suggest that HIV-2 and SIV_mac_ both originated as a result of the cross-species transmission of SIV_smm_ from sooty mangabeys to humans and from sooty mangabeys to rhesus macaques respectively [32],[33],[34],[35],[36]. The common origin of these viruses has therefore led to speculation that SIV may also use its envelope glycoprotein to overcome restriction by tetherin.

As a consequence of the ongoing evolutionary conflict between viral pathogens and host cell defenses, many antiviral restriction factors have acquired amino acid differences that are important host-range determinants of viral infection [37],[38]. TRIM5α was first identified as the post-entry block to HIV-1 infection of cells from rhesus macaques [39], and differences in the B30.2 (SPRY) domain of TRIM5α are now recognized as important host-range determinants of the primate lentiviruses [40],[41],[42],[43]. Likewise, species-specific differences in the APOBEC family of cytidine deaminases are significant host-range determinants for diverse retroviruses [44],[45],[46],[47]. A single amino acid difference in simian versus human APOBEC3G accounts for the inability of HIV-1 Vif to overcome this restriction in Old World monkeys [48],[49],[50]. Comparison of the amino acid sequences of tetherin orthologues from different mammalian species suggests that tetherin may also be rapidly evolving under positive selection in response to viral pathogens [13],[51]. Indeed, recent studies have now demonstrated that the ability of Vpu to antagonize tetherin is species-specific [51],[52].

Here we show that SIV Nef overcomes restriction by rhesus macaque and sooty mangabey tetherin, but not human tetherin. Conversely, HIV-1 Vpu counteracts restriction by human tetherin, but is ineffective against macaque or mangabey tetherin. The specificity of SIV Nef for rhesus tetherin mapped to a four amino acid sequence in the cytoplasmic domain that is missing from the human protein, whereas the specificity of HIV-1 Vpu for human tetherin mapped to amino acid differences in the trans-membrane domain. Thus, similar to TRIM5α and APOBEC3G, species-specific differences in tetherin may influence the host-range of primate lentiviruses.

## Results

### SIV Nef counteracts restriction by rhesus tetherin

**Figure 1:**
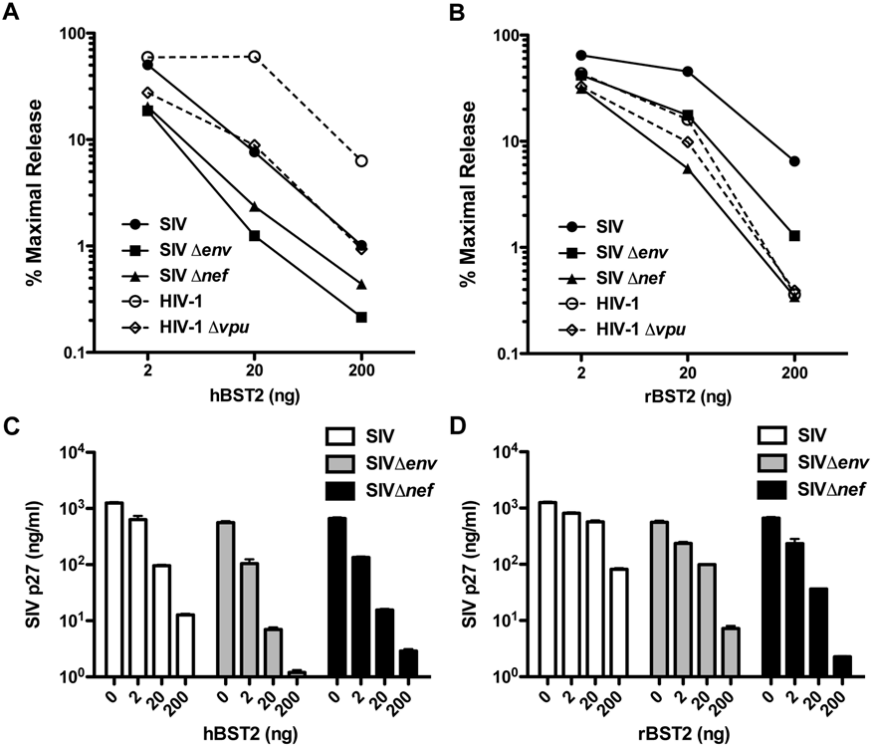
Identification of Nef as the viral gene product of SIV that counteracts restriction by tetherin. SIV, SIV Δ*env*, SIV Δ*nef*, HIV-1 and HIV-1 Δ*vpu* were tested for particle release in the presence of human (hBST2) and rhesus (rBST2) tetherin. Virus production as a percentage of maximal particle release in the absence of tetherin is shown at increasing amounts of plasmid DNA for hBST2 (A) and rBST2 (B). The mean and standard deviation (error bars) for total p27 release is also shown for wild-type SIV, SIV Δ*env* and SIV Δ*nef* at the indicated amounts of DNA for hBST2 (C) and rBST2 (D). 293T cells were transfected in duplicate with proviral DNA constructs based on SIV_mac_239 and HIV-1 NL4-3 together with expression constructs for either hBST2 or rBST2 (0, 2, 20 and 200 ng). Differences in the amount of plasmid DNA in each transfection were compensated by the addition of empty vector (pcDNA3). After 48 hours, the amount of virus released into the cell culture supernatant was measured by HIV-1 p24 and SIV p27 antigen-capture ELISA.

Deletion mutants of SIV were tested for particle release in cells expressing tetherin to identify the viral gene product(s) that oppose this restriction factor. Wild-type and *vpu*-deleted strains of HIV-1 were also tested as controls. These assays were performed by transfection of 293T cells with full-length HIV-1 NL4-3 and SIV_mac_239 proviral DNA mutants together with expression constructs for either human or rhesus tetherin (hBST2 or rBST2). 293T cells were selected for these assays since previous studies have shown that these cells do not express endogenous tetherin in the absence of IFNα induction [1],[11]. Virus release was measured by HIV-1 p24 and SIV p27 antigen-capture ELISA at 0, 2, 20 and 200 ng of plasmid DNA for each tetherin expression construct (Fig. 1). Virion release for each strain was then compared as a percentage of maximal particle release in the absence of tetherin.

Consistent with previous reports, HIV-1 *Δvpu* was more sensitive to restriction by human tetherin than wild-type HIV-1 (Fig. 1A) [1],[2]. In the case of SIV, particle release for wild-type virus, and for each of the deletion mutants, was inhibited to a similar or greater extent than for HIV-1 *Δvpu* (Fig. 1A). Thus, SIV appeared to be unable to counteract restriction by human tetherin. We therefore compared virion production in the presence of rhesus tetherin. Wild-type SIV was considerably more resistant than HIV-1 to restriction by rhesus tetherin (Fig. 1B). Moreover, both wild-type and *vpu*-deleted HIV-1 exhibited similar susceptibility to rhesus tetherin (Fig. 1B). Thus, HIV-1 also appeared to be unable to counteract restriction by rhesus tetherin. The inherent susceptibility of HIV-1 to rhesus tetherin and SIV to human tetherin suggested that the countermeasures used by these viruses were species-specific.

In cells expressing rhesus tetherin, the greatest reduction in particle release was observed for SIV *Δnef*. Compared to wild-type SIV, SIV *Δnef* was inhibited 2- to 22-fold in the presence of rBST2 (Fig. 1B). A lesser 1.5- to 5-fold reduction was also observed for SIV *Δenv*. However, similar reductions in virus release were also observed for SIV *Δenv* in the presence of hBST2 (Fig. 1A), and in the absence of tetherin (Fig. 1C & D). Thus, it is unclear to what extent these differences may reflect additional non-specific effects of envelope on virus assembly and release. SIV mutants with deletions in *vpr* and *vpx* were also tested, but elimination of these genes did not significantly impair virus release in the presence of either human or rhesus tetherin (data not shown). These experiments therefore pointed to a role for the SIV Nef protein in overcoming restriction by rhesus tetherin.

**Figure 2:**
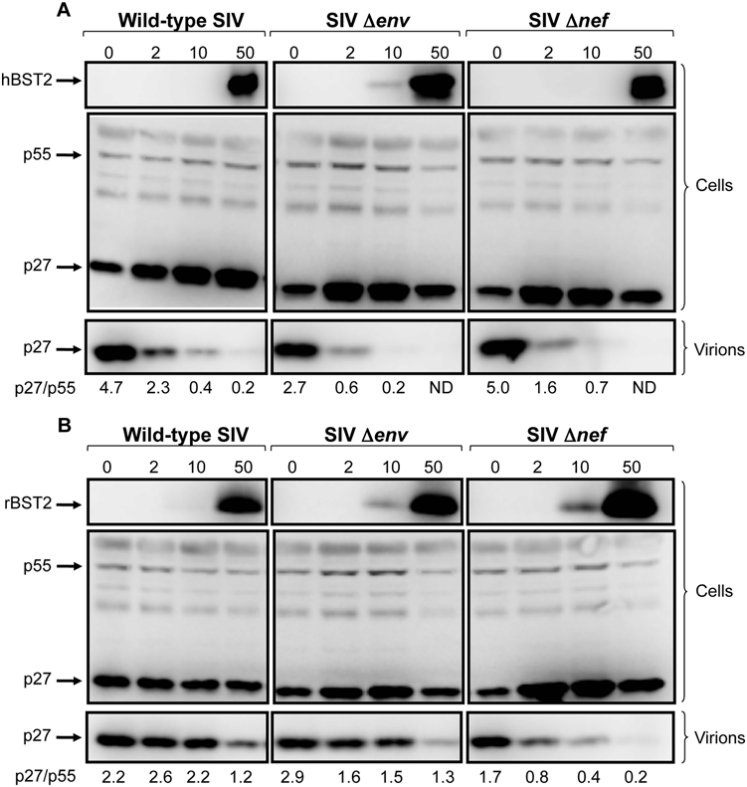
Dose-dependent reduction in the accumulation of SIV p27 in the cell culture supernatant relative to p55 Gag in cells at increasing expression levels of tetherin. The accumulation of SIV p27 capsid in the cell culture supernatant relative to p55 Gag in cell lysates was compared for wild-type SIV, SIV *Δenv* and SIV Δ*nef* at increasing expression levels of human and rhesus tetherin. 293T cells were transfected with proviral DNA for wild-type SIV, SIV *Δenv* or SIV Δ*nef* together with 0, 2, 10 and 50 ng of plasmid DNA for hBST2 (A) or rBST2 (B). Forty-eight hours post-transfection, virus was recovered from the culture supernatant by centrifugation and cell lysates were prepared. Proteins were separated on a 10% SDS-polyacrylamide gel, transferred to a PVDF membrane and probed with monoclonal antibodies to p27/p55 Gag (183-H12-5C) and to BST2 (HM1.24). The blots were then probed with an HRP-conjugated goat anti-mouse secondary antibody, developed in chemiluminescent substrate and visualized using a Fujifilm Image Reader LAS 3000. Band intensities for p27 in virions and p55 in cell lysates were determined using the Image J Software (Rasband, W.S., Image, U.S. NIH, Bethesda, MD, http://rsb.info.nih.gov/ij, 1997–2008) and compared as p27/p55 ratios. ‘ND’ indicates that the p27 band was below the limit of detection.

To confirm that these results were due to the effects of tetherin on particle release rather than on viral protein expression, we compared levels of the SIV p55 Gag protein in cells to the accumulation of p27 capsid in cell culture supernatant by western blot analysis. For wild-type SIV, SIV *Δenv* and SIV *Δnef*, similar amounts of the p55 Gag protein were detected in cell lysates over a 0 to 50 ng range of plasmid DNA for both hBST2 and rBST2 (Fig. 2). In cells expressing human tetherin, there was a dose-dependent decrease in the accumulation of p27 recovered from the cell culture supernatant for each of these viruses (Fig. 2A & B). A similar reduction in p27 was also observed for SIV *Δnef* at increasing levels of rhesus tetherin (Fig. 2B). These reductions in supernatant were accompanied by increases in cell-associated p27. However, p27 levels in supernatant for wild-type SIV and SIV *Δenv* were less sensitive to the effects of rhesus tetherin, as reflected by more consistent p27/p55 (supernatant/cell lysate) ratios (Fig. 2B). Although a partial reduction in p27 was observed for wild-type SIV and SIV *Δenv* under conditions of overexpression, suggesting that the ability to overcome restriction was saturable, the p27 band remained detectable even at 50 ng of the rBST2 expression construct (Fig. 2B). Thus, these results support a role for the SIV Nef protein in opposing the inhibition of vrus release by tetherin.

### Species-specificity of SIV Nef and HIV-1 Vpu in counteracting restriction by tetherin

**Figure 3:**
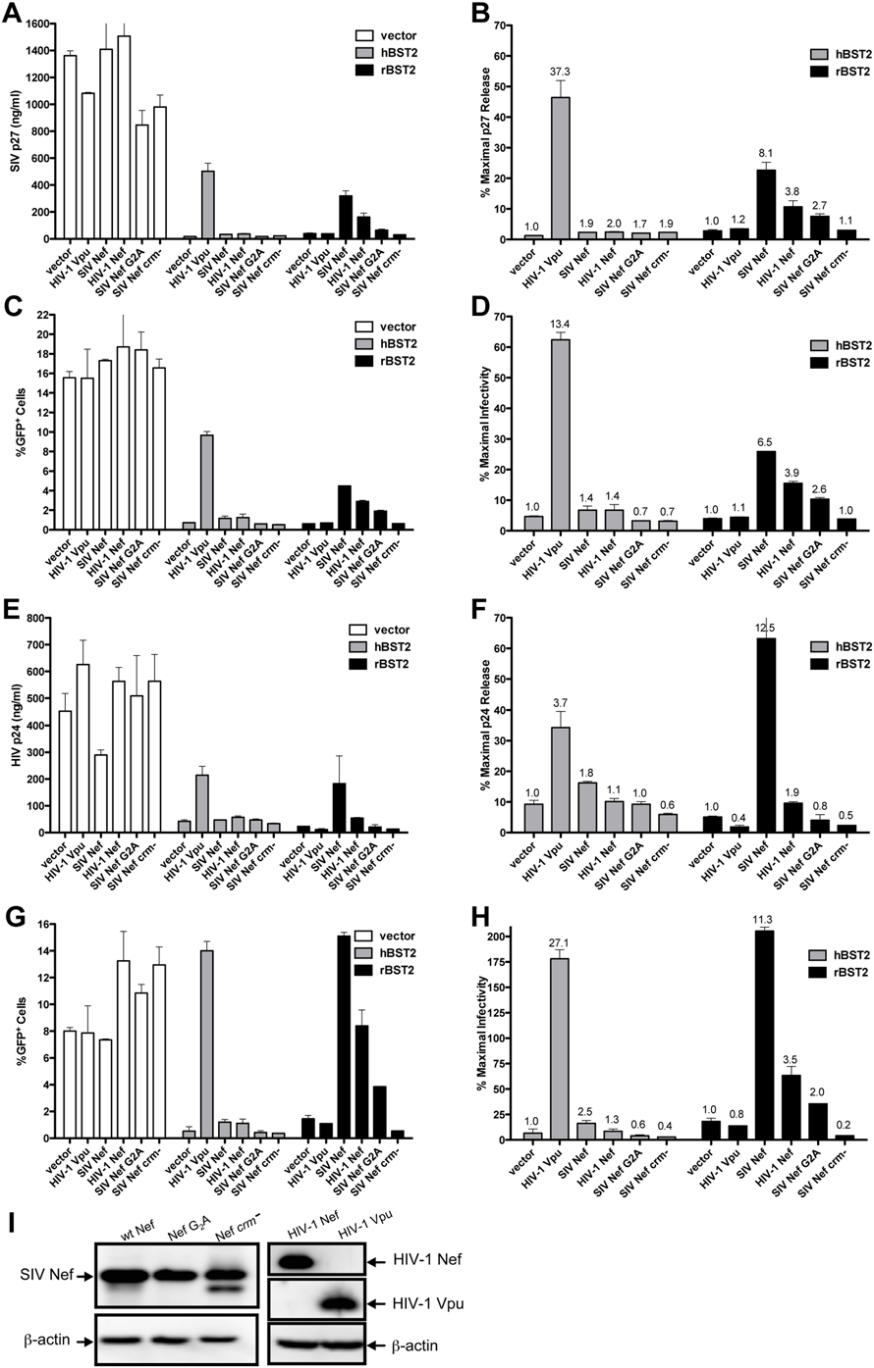
Species-specificity of HIV-1 Vpu and SIV Nef in counteracting restriction by human and rhesus tetherin. HIV-1 Vpu and SIV Nef were tested for the ability to rescue virus release in *trans* for SIV Δ*nef* and a *vpu*-deficient strain of HIV-1 in the presence of human and rhesus tetherin. 293T cells were transfected in duplicate with 100 ng of proviral DNA for SIV Δ*nef* (A–D) or HIV-1 HXB2 (E–H), 50 ng of DNA for hBST2 or rBST2, and 100 ng of DNA for either HIV-1 Vpu, SIV Nef, SIV Nef G_2_A, SIV Nef *crm^−^* or HIV-1 Nef. G_2_A and *crm^−^* represent amino acid substitutions in the myristoylation site and the cholesterol recognition motif (L_129_R, Y_133_A and Y_134_A) of SIV Nef respectively. Vector controls included pcDNA3 for the tetherin expression constructs and pCGCG for the Nef expression constructs. Forty-eight hours post-transfection, the amount of virus released into the cell culture supernatant was measured by SIV p27 (A) or HIV-1 p24 (E) antigen-capture ELISA, and by infectivity on GHOST X4/R5 cells (C,G). To control for variability due to tetherin-independent effects of Nef and Vpu on particle release, the data are also expressed as percent maximal release in the absence of tetherin (B,D,F,H). The values above each bar represent the fold-increase in virus release compared to the empty vector control. The expression of wild-type SIV Nef, Nef G_2_A, Nef *crm-*, HIV-1 Nef and HIV-1 Vpu were verified by western blot analysis (I).

The capacity of SIV Nef and HIV-1 Vpu to restore particle release in *trans* in the presence of human and rhesus tetherin was tested to further investigate the species-specificity of these viral proteins. Virus release for SIV *Δnef* and HIV-1 HXB2, a *vpu*-deficient strain of HIV-1, was measured by antigen-capture ELISA, and by infectivity on GHOST cells expressing CXCR4 and CCR5 (GHOST X4/R5 cells) [53]. The results of these assays are presented as total p24/p27 release (Fig. 3A & E), or as the frequency of infected cells following inoculation of GHOST X4/R5 cells with cell culture supernatant (Fig. 3C & G). The data are also presented as the percentage of maximal release in the absence of tetherin to control for additional effects of Nef and Vpu on particle release independent of their activity against tetherin (Fig. 3B, D, F & H). In each case, Vpu restored particle release in cells expressing human tetherin, and SIV Nef restored particle release in cells expressing rhesus tetherin, but not vice versa (Fig. 3A–H). Hence, these results confirmed the species-specific activity of both HIV-1 Vpu and SIV Nef in opposing restriction by tetherin.

In cells expressing rhesus tetherin, SIV Nef enhanced p27 release for SIV *Δnef* 8.1-fold and p24 release for HIV-1 HXB2 12.5-fold (Fig. 3B & F). Similar corresponding increases were also observed in the amount of infectious virus (Fig. 3D & H). To verify that the activity of SIV Nef in counteracting rhesus tetherin was not the result of protein overexpression, Nef mutants with amino acid substitutions at positions predicted to broadly disrupt functional activities of Nef, without altering protein folding or stability, were also tested [54]. A glycine to alanine substitution in the N-terminal myristoylation site (G_2_A), which disrupts the localization of the protein to cellular membranes, significantly reduced the ability of Nef to facilitate virus release in the presence of rhesus tetherin (Fig. 3). Likewise, amino acid substitutions in a putative cholesterol recognition motif (*crm^−^*; L_129_R, Y_133_A and Y_134_A), previously reported to disrupt the association of HIV-1 Nef with cholesterol-rich lipid microdomains [54], completely abolished this activity of the SIV Nef protein (Fig. 3). Western blot analysis confirmed that the G_2_A and *crm* ^−^ mutants were expressed at similar levels to the wild-type SIV Nef protein, although a minor band representing a potential degradation product of the *crm^−^* mutant was observed (Fig. 3I). These results indicate that the activity of Nef in counteracting rhesus tetherin does not simply reflect a non-specific effect of protein overexpression. These results further suggest that the ability of SIV Nef to overcome restriction by rhesus tetherin may depend on its localization to cellular membranes.

We also observed a partial effect of HIV-1 Nef on virus release. HIV-1 Nef resulted in a 3.8-fold increase in SIV p27 release and a 1.9-fold increase in HIV-1 p24 release in cells expressing rhesus tetherin (Fig. 3B & F). Consistent with the previously documented role for Nef in infectivity enhancement [55],[56],[57],[58],[59], this effect was greater (3.5-fold) when HIV-1 release was measured by infectivity on GHOST X4/R5 cells rather than by p24 ELISA (Fig. 3F & H). However, HIV-1 Nef did not significantly increase the release of either SIV *Δnef* or HIV-1 HXB2 in cells expressing human tetherin. Although HIV-1 Nef activity against rhesus tetherin may seem at odds with the adaptation of HIV-1 for replication in humans, it is conceivable that conserved sequences in Nef, possibly maintained by virtue of their role in other functional activities of the protein, contribute to a basal level of HIV-1 Nef activity against rhesus tetherin.

In addition to facilitating particle release, Vpu also enhanced HIV-1 infectivity. While Vpu increased p24 release by 3.7-fold, infectivity was increased by 27.1-fold in the presence of human tetherin (Fig. 3F & H). This represents a 5-fold increase in HIV-1 particle infectivity. In contrast to the infectivity enhancement afforded by Nef, this effect was not observed in the absence of tetherin or in cells expressing rhesus tetherin (Fig. 3G). Furthermore, Vpu did not increase the infectivity of SIV *Δnef* (Fig. 3B & D). Thus, the infectivity enhancement afforded by Vpu appears to be directly related to its role in counteracting human tetherin and to be specific for HIV-1. These results are consistent with previous observations [1],[2],[52], and suggest that Vpu may have an under appreciated role in enhancing the infectivity of HIV-1 that contributes to its activity in overcoming restriction by tetherin.

### The SIV envelope glycoprotein does not rescue virion release in the presence of human or rhesus tetherin

**Figure 4:**
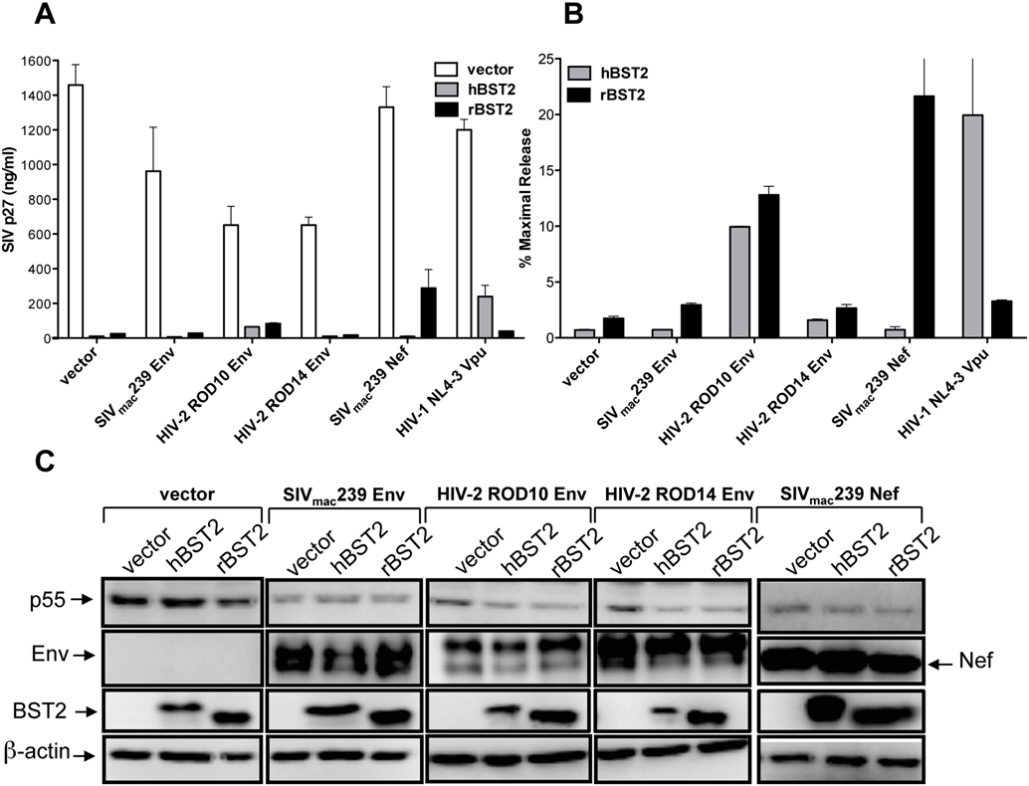
Expression of the SIV envelope glycoprotein does not rescue virus release. The envelope glycoproteins of SIV_mac_239, HIV-2 ROD10 and HIV-2 ROD14 were tested for the ability to rescue virus release for SIV *ΔenvΔnef* in cells expressing human and rhesus tetherin. 293T cells were transfected with proviral DNA for SIV *ΔenvΔnef*, an expression construct for hBST2 or rBST2, and an expression construct for either SIV_mac_239 Env, HIV-2 ROD10 Env, HIV-2 ROD14 Env, SIV_mac_239 Nef or HIV-1 NL4-3 Vpu. The mean and standard deviation (error bars) are shown for total p27 release (A) and for percent maximal release (B). (C) Protein expression was confirmed for SIV Env, HIV-2 Env, SIV Nef, SIV p55 Gag and BST2 by western blot analysis of cell lysates. SIV Env was detected with a monoclonal antibody to SIV gp120 (KK42) and HIV-2 Env was detected with rabbit antisera to HIV-2 ST gp120. The SIV Nef protein was detected with the monoclonal antibody 17.2. SIV p55 Gag, BST2 and β-actin were detected with the monoclonal antibodies 183-H12-5C, HM1.24 and C4 respectively. Following incubation with an appropriate HRP-conjugated secondary antibody, the blots were developed in chemiluminescent substrate and visualized using a Fujifilm Image Reader LAS 3000.

Since deletion of the *env* gene resulted in a modest increase in the sensitivity of SIV to both human and rhesus tetherin, and the envelope glycoproteins of certain HIV-2 isolates have been shown to have Vpu-like activity [28],[29],[30],[31], we further examined the ability of the SIV envelope glycoprotein to oppose restriction by tetherin. The relative contribution of SIV Env versus SIV Nef to particle release was compared for an *env*- and *nef*-deleted strain of SIV (SIV *ΔenvΔnef*) in cells expressing human and rhesus tetherin. We also tested the envelope glycoproteins of two closely related HIV-2 isolates; one with Vpu-like activity, HIV-2 ROD10 Env, and one without, HIV-2 ROD14 Env [30],[31]. Consistent with previous reports [30],[31], HIV-2 ROD10 Env enhanced virus release in cells expressing both human and rhesus tetherin, whereas HIV-2 ROD14 Env did not (Fig. 4). These results suggest, perhaps not surprisingly, that the HIV-2 ROD10 envelope glycoprotein has a role in counteracting viral inhibition by tetherin, but unlike Vpu or Nef, this activity is not species-specific. However, similar to HIV-2 ROD14 Env, SIV Env failed to rescue virus release in cells expressing either human or rhesus tetherin (Fig. 4). In contrast, SIV Nef increased p27 release by more than 12-fold in cells expressing rhesus tetherin (Fig. 4). The inability to detect a significant increase in virus release under conditions of SIV Env overexpression further suggests that the SIV envelope glycoprotein does not play a major role in counteracting restriction by tetherin.

### SIV Nef downregulates rhesus tetherin from the cell surface

**Figure 5:**
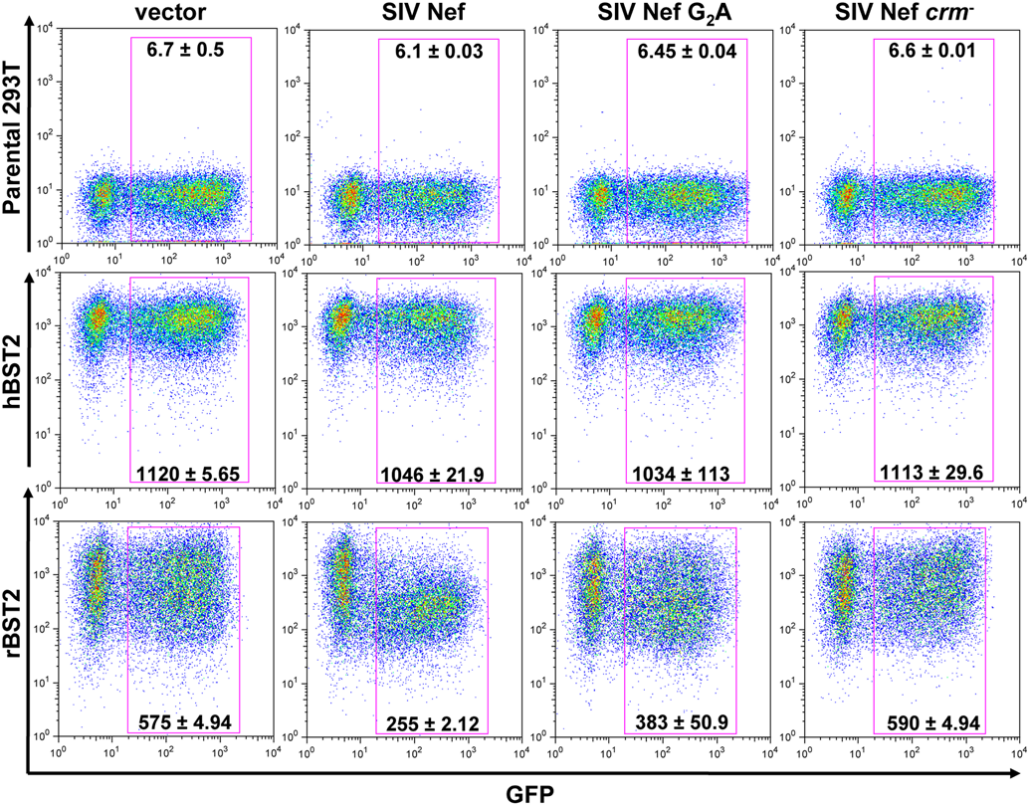
SIV Nef downregulates rhesus tetherin, but not human tetherin. The ability of Nef to downmodulate tetherin from the cell surface was assessed by transfecting stable 293T cell lines expressing HA-tagged human or rhesus tetherin (hBST2 or rBST2) with bicistronic constructs expressing Nef and GFP. SIV Nef, SIV Nef G_2_A and SIV Nef *crm^−^* were expressed from the same mRNA transcript as GFP using pCGCG constructs in which expression of the *GFP* reporter gene was driven from an internal ribosomal entry site downstream of *nef*. Cells were also transfected with pCGCG without *nef* as an empty vector control. Twenty-four hours after transfection, the cells were stained with an anti-HA monoclonal antibody followed by an APC-conjugated, donkey anti-mouse polyclonal antibody and analyzed by flow cytometry. The values indicated in each plot represent the mean fluorescence intensity and standard deviation of HA/BST2 staining on GFP^+^ cells for duplicate transfections.

Since Nef is known to downregulate a number of proteins from the cell surface [60], and Vpu has been shown to downregulate tetherin [2], we asked whether SIV Nef could also downregulate rhesus macaque tetherin. To address this question, stable 293T cell lines expressing human and rhesus tetherin with HA tags in their extracellular domains were transfected with bicistronic constructs that co-express Nef together with green fluorescent protein (GFP). The extent of tetherin downmodulation was determined by comparing the mean fluorescence intensity (MFI) of HA-staining on GFP^+^ cells transfected with the empty vector to the MFI of HA-staining on GFP^+^ cells expressing Nef. Consistent with the species-specific activity of Nef in mediating virus release, SIV Nef downregulated rhesus tetherin, but did not downregulate human tetherin (Fig. 5). Moreover, mutations in the myristoylation site and putative cholesterol recognition motif that impaired the ability of Nef to facilitate virus release also disrupted its ability to downregulate rhesus tetherin (Fig. 5). Thus, similar to Vpu [2], SIV Nef appears to specifically downmodulate cell-surface expression of rhesus tetherin.

### Interferon induces tetherin and inhibits the release of *nef*-deleted SIV from an infected rhesus macaque cell line

**Figure 6:**
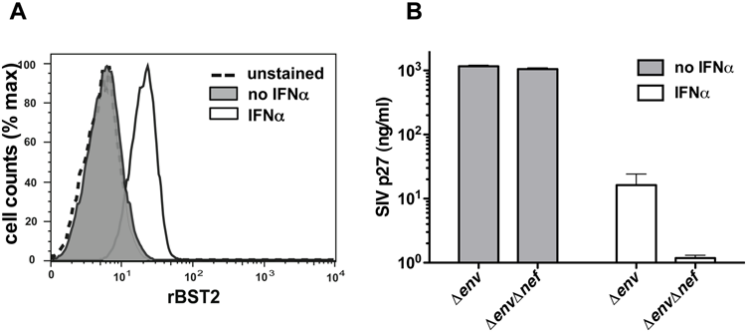
Interferon induces tetherin and inhibits the release of *nef*-deleted SIV from an infected rhesus macaque cell line. The rhesus macaque sMAGI cell line was cultured in the presence or absence of IFNα, and virus release was compared following infection with VSV G-pseudotyped SIV *Δenv* versus SIV *ΔenvΔnef*. (A) Treatment with IFNα upregulated the expression of tetherin on the cell surface. Cells were cultured in medium with or without 1000 U/ml IFNα, and the expression of tetherin was assessed by flow cytometry. (B) Deletion of the SIV *nef* gene significantly impaired virus release from infected cells in the presence of IFNα. Seventy-two hours after infection with VSV G-pseudotyped SIV *Δenv* and SIV *ΔenvΔnef* (50 ng/ml p27 eq. each), the amount of p27 released into the cell culture supernatant was determined by antigen capture ELISA.

To determine if deletion of the *nef* gene also impaired virus release from an SIV-infected rhesus macaque cell line expressing physiological levels of tetherin, sMAGI cells, cultured in the presence and absence of IFNα, were infected with VSV G-pseudotyped SIV *Δenv* versus SIV *ΔenvΔnef*. While this cell line did not express dectable levels of tetherin in the absence of interferon, treatment with IFNα resulted in the upregulation of tetherin on the cell surface (Fig. 6A). Although IFNα significantly impaired virus release for both SIV *Δenv* and SIV *ΔenvΔnef*, we observed a much greater reduction in particle release for SIV *ΔenvΔnef*. Compared to SIV *Δenv*, p27 release for SIV *ΔenvΔnef* was reduced an additional 13.8-fold in IFNα-treated cells (Fig. 6B). Hence, deletion of the SIV *nef* gene significantly impaired virus release from infected cells in the presence of interferon.

### The specificity of SIV Nef for rhesus tetherin maps to a four amino acid sequence in the N-terminus of the molecule that is not present in human tetherin

Since Nef is a membrane-associated cytosolic protein, we reasoned that it could potentially associate with the cytoplasmic domain of tetherin. We therefore exchanged the cytoplasmic domains of human and rhesus tetherin and tested these recombinants for susceptibility to SIV Nef. SIV Nef restored particle release when the cytoplasmic domain of rhesus tetherin was fused to the transmembrane and extracellular domain of human tetherin, but not vice versa, indicating that the ability of SIV Nef to antagonize rhesus tetherin was dependent on sequences in the cytoplasmic domain (data not shown).

**Figure 7:**
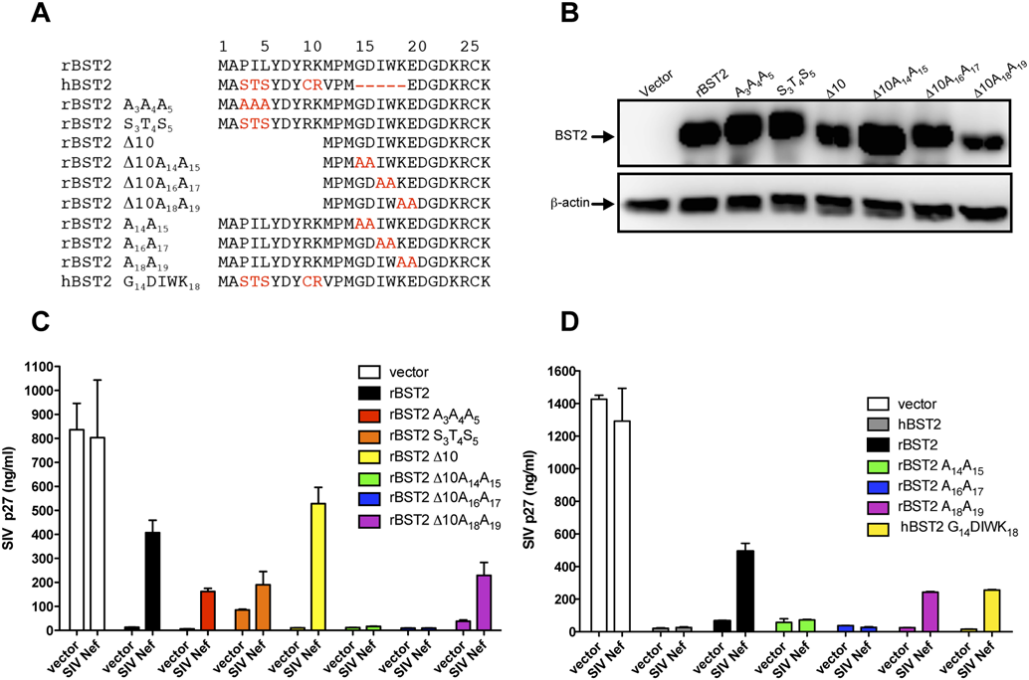
Identification of the residues in rhesus tetherin required for recognition by SIV Nef. (A) Amino acid substitutions were introduced into full-length rBST2 and a deletion mutant lacking the first ten amino acids (rBST2 Δ10) at positions that differ from hBST2. The G_14_DIWK_18_ motif of rBST2 was also introduced into hBST2 (hBST2 G_14_DIWK_18_). Dashes represent sequence gaps, and positions that differ from wild-type rBST2 are indicated in red. (B) Expression of each of the rBST2 mutants tested in (C) was confirmed by western blot analysis of transfected 293T cell lysates. (C,D) SIV Nef was tested for the ability to rescue virus release for SIV *Δnef* in cells expressing each of the rBST2 and hBST2 mutants shown in (A). Transfection and assay conditions were the same as previously described.

Amino acid substitutions were therefore introduced into the cytoplasmic domain of rhesus tetherin at positions that differed from human tetherin to further define the sequences required for recognition by SIV Nef (Fig. 7A). Western blot analysis confirmed the expression of each of these mutants, although there appeared to be some variability in protein detection (Fig. 7B). Repeated analysis of the same samples indicated that these differences in band intensity were in part due to inconsistencies in the detergent solubility of this transmembrane protein, and thus do not reflect quantitative levels of protein expression. Substitution of amino acids 3–5 with the corresponding residues of human tetherin (S_3_T_4_S_5_), or with alanine residues (A_3_A_4_A_5_), diminished, but did not eliminate, the effect of Nef on particle release (Fig. 7C). Furthermore, deletion of the first 10 amino acids (Δ10) did not affect the ability of Nef to facilitate virus release (Fig. 7C). These results therefore implicated the remaining 16-residue membrane proximal region in recognition by the SIV Nef protein.

The most conspicuous difference between rhesus and human tetherin in this region was a five amino acid sequence that is missing from the human molecule. Alanine substitutions were introduced into this region in the context of the Δ10 truncation mutant to investigate the possibility that these sequences accounted for the specificity of Nef (Fig. 7A). Pair wise alanine substitutions at positions 14–15 (A_14_A_15_) and 16–17 (A_16_A_17_) eliminated the ability of Nef to enhance virus release, while adjacent substitutions at positions 18–19 (A_18_A_19_) did not (Fig. 7C). These substitutions were also introduced into full-length rhesus tetherin, and similar to the truncation mutants, the A_14_A_15_ and A_16_A_17_ substitutions abrogated the ability of Nef to rescue virion release, while the A_18_A_19_ substitions did not (Fig. 7D). Moreover, the introduction of the G_14_DIWK_18_ motif from rhesus tetherin into human tethern resulted in a gain-of-function for susceptibility to Nef (Fig. 7D). Thus, a four amino acid sequence, G_14_DIW_17_, that is present in the cytoplasmic domain of rhesus tetherin, but not human tetherin, accounts for the species-specific activity of SIV Nef against rhesus tetherin.

### The specificity of HIV-1 Vpu maps to the membrane-spanning domain of human tetherin

**Figure 8:**
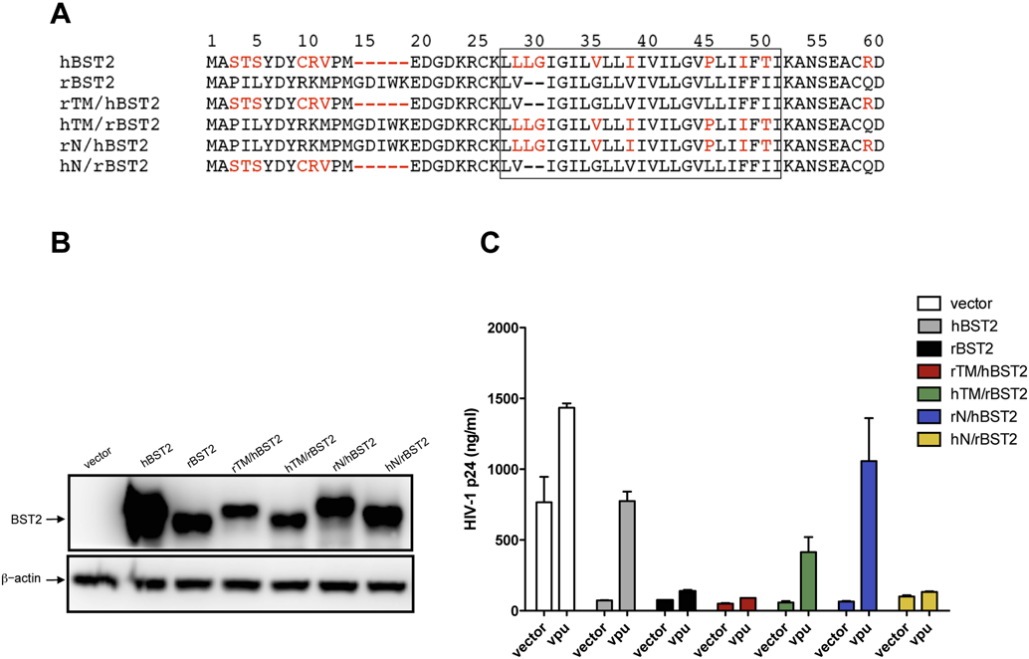
The sequences required for Vpu recognition of human tetherin map to the transmembrane domain. (A) Sequences corresponding to the cytoplasmic domain (N) and transmembrane domain (TM) of human and rhesus tetherin (hBST2 and rBST2) were exchanged to generate the chimeric fusion proteins rTM/hBST2, hTM/rBST2, rN/hBST2 and hN/rBST2. Amino acid sequences corresponding to the predicted transmembrane domains are boxed, dashes represent sequence gaps, and positions in hBST2 that differ from rBST2 are indicated in red. (B) The expression of each of these recombinant proteins was verified by western blot analysis. (C) Vpu was tested for the ability to rescue p24 release for HIV-1 HXB2 in 293T cells expressing hBST2, rBST2, rTM/hBST2, hTM/rBST2, rN/hBST2 and hN/rBST2. Transfection and assay conditions were the same as described previously.

Recombinants between human and rhesus tetherin were also tested to identify sequence differences that accounted for the species-specificity of Vpu. Sequences coding for the cytoplasmic domains were exchanged to generate rN/hBST2 and hN/rBST2 and sequences coding for the transmembrane domains were exchanged to generate rTM/hBST2 and hTM/rBST2 (Fig. 8A). The expression of each of these chimeric fusion proteins was verified by western blot analysis (Fig. 8B). These recombinants were then tested for the ability to inhibit virus release in the presence and absence of Vpu.

Vpu restored particle release for the rN/hBST2 chimera, in which the cytoplasmic domain of human tetherin was exchanged with the cytoplasmic domain of rhesus tetherin, but not for the reciprocal hN/rBST2 chimera (Fig. 8C). Thus, the specificity of Vpu was not affected by exchanging the cytoplasmic domains of hBST2 and rBST2. However, susceptibility to Vpu was reversed by exchanging their transmembrane domains. Replacement of the transmembrane domain of human tetherin with the corresponding sequences from rhesus tetherin (rTM/hBST2) abrogated the ability of Vpu to facilitate virus release (Fig. 8C). For the reciprocal exchange, replacement of the transmembrane domain of rhesus tetherin with the transmembrane domain of human tetherin (hTM/rBST2) resulted in a gain-of-function for Vpu-mediated particle release (Figs 8C). Hence, these experiments mapped the specificity of Vpu to the transmembrane domain of human tetherin. Since Vpu is also an integral membrane protein, and membrane-spanning sequences at its N-terminus are required for the ability to enhance virion release [61],[62],[63], these results suggest that the mechanism by which Vpu antagonizes tetherin may involve interactions between the transmembrane domains of these proteins.

### Nef alleles of SIV_smm/mac_, HIV-2 and HIV-1 counteract rhesus macaque and sooty mangabey tetherin, but are generally ineffective against human tetherin

**Figure 9:**
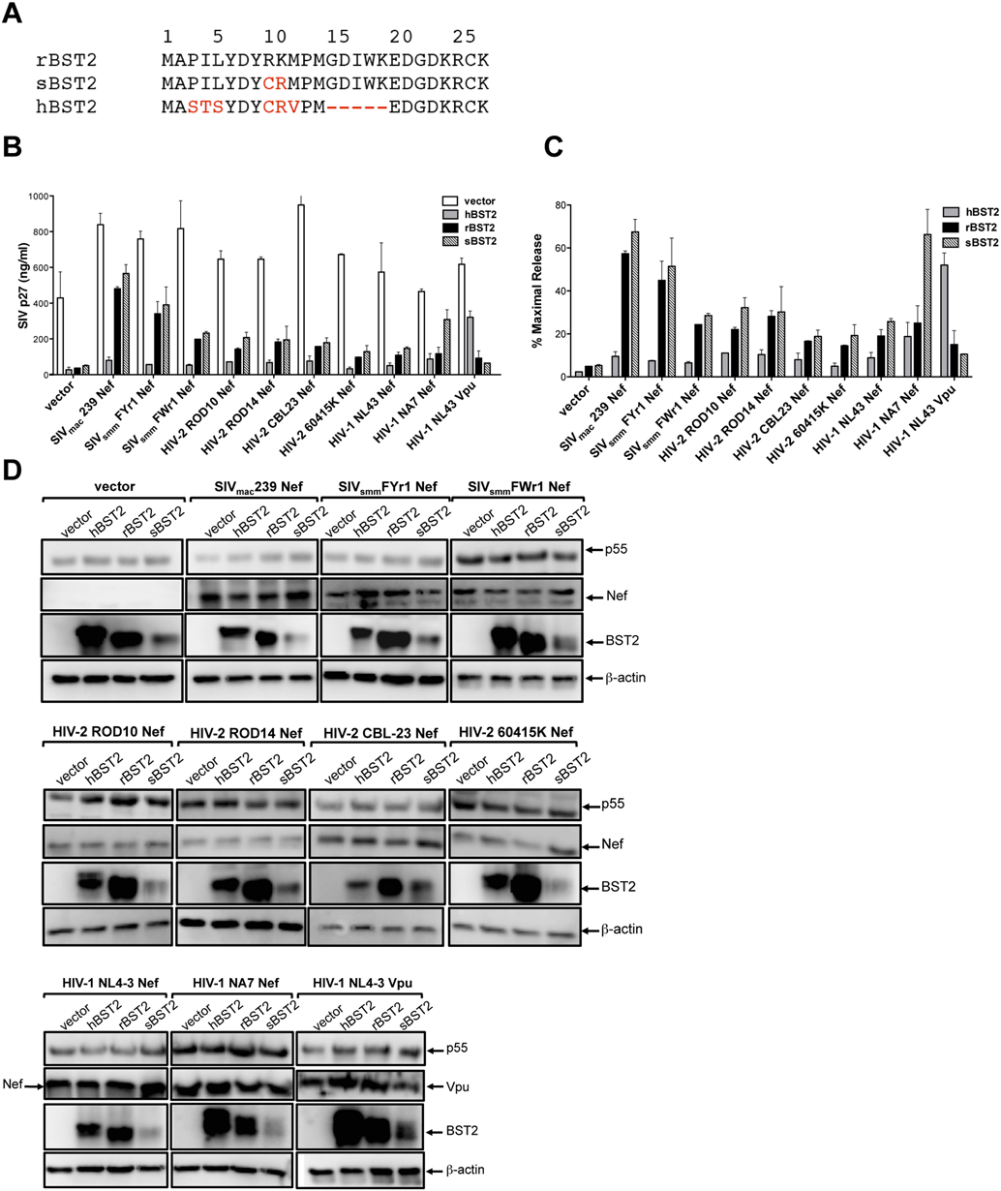
Nef alleles of SIV_smm/mac_, HIV-2 and HIV-1 counteract rhesus macaque and sooty mangabey tetherin, but not human tetherin. Nef alleles of SIV_smm/mac_, HIV-2 and HIV-1 were tested for the ability to rescue particle release for SIV *Δnef* in the presence of human tetherin (hBST2), rhesus macaque tetherin (rBST2) and sooty mangabey tetherin (sBST2). (A) The amino acid sequences corresponding to the cytoplasmic domains of hBST2, rBST2 and sBST2 are shown. Dashes represent sequence gaps and residues that differ from rBST2 are indicated in red. The mean and standard deviation (error bars) for total p27 release (B) and for percent maximal release (C) are shown for the indicated Nef alleles of SIV_smm/mac_, HIV-2 and HIV-1 in the presence of hBST2, rBST2 and sBST2. (D) Protein expression was confirmed for SIV p55 Gag, BST2, HIV-1 Vpu and for each of the Nef alleles by western blot analysis of 293T cell lysates. The Nef proteins of SIV_mac_239 and SIV_smm_ (FYr1 and FWr1) were detected using plasma pooled from SIV-infected rhesus macaques and SIV-infected sooty mangabeys respectively. The Nef proteins of HIV-2 ROD10, ROD14, CBL-23 and 60415K were detected using plasma pooled from HIV-2-infected individuals. The Nef proteins of HIV-1 NL4-3 and NA7 were detected using polyclonal rabbit antisera. SIV p55 Gag, BST2 and β-actin were detected with the monoclonal antibodies 183-H12-5C, HM1.24 and C4. Following incubation with an appropriate HRP-conjugated secondary antibody, the blots were developed in chemiluminescent substrate and visualized using a Fujifilm Image Reader LAS 3000.

Nef alleles of SIV_smm/mac_, HIV-2 and HIV-1 were tested for the ability to rescue particle release in cells expressing rhesus macaque, sooty mangabey and human tetherin. Similar to SIV_mac_239 Nef, Nef alleles of two primary SIV_smm_ isolates, SIV_smm_FYr1 and SIV_smm_FWr1, enhanced virus release in the presence of rhesus macaque tetherin (Fig. 9B & C). These alleles also enhanced virus release from cells expressing sooty mangabey tetherin to a similar extent as SIV_mac_239 Nef (Fig. 9B & C). These observations are supported by western blots showing consistent levels of Nef and p55 Gag expression in each set of transfections (Fig. 9D). The bands for human, rhesus macaque and sooty mangabey tetherin were more variable, but nevertheless provided qualitative verification of protein expression (Fig. 9D). These results therefore suggest that the ability of Nef to oppose tetherin was likely retained upon cross-species transmission of SIV_smm_ from sooty mangabeys to rhesus macaques.

Although somewhat less efficiently, Nef alleles of HIV-1 and HIV-2 also rescued virus release in the presence of rhesus macaque and sooty mangabey tetherin (Fig. 9B & C). Thus, the ability of Nef to counteract tetherin from these Old World monkeys appears to be broadly conserved among the primate lentiviruses. However, with the exception of HIV-1 NA7 Nef, none of the Nef alleles tested appreciably restored virus release in cells expressing human tetherin. Interestingly, HIV-1 NA7 Nef was particularly effective at facilitating virus release in the presence of sooty mangabey tetherin (Fig. 9B & C). Since the cytoplasmic domains of rhesus macaque and sooty mangabey tetherin only differ by two amino acids C_9_R_10_, and these residues are present in both the human and sooty mangabey molecules (Fig. 9A), it is conceivable that adaptation of HIV-1 NA7 for partial activity against human tetherin may have resulted in greater activity against the sooty mangabey molecule.

## Discussion

Tetherin (BST2, CD317 or HM1.24) was recently identified as an interferon-inducible restriction factor that interferes with the detachment of HIV-1 from infected cells in the absence of the Vpu accessory protein [1],[2]. While this factor appears to have broad activity against diverse retroviruses [1],[2],[27], as well as other families of enveloped viruses [25],[26],[27], only two phylogenetic groups of primate lentiviruses, HIV-1/SIV_cpz_ and SIV_gsn/mon/mus_, are known to express Vpu [21],[22],[23],[24]. Lentiviruses of the HIV-2/SIV_smm/mac_ lineage do not have a *vpu* gene. Thus, understanding how these viruses overcome restriction by tetherin is of significant evolutionary interest and may have important implications to our understanding of the host-range specificity of the primate lentiviruses. We therefore set out to determine how SIV overcomes this restriction in the rhesus macaque, an important animal model for lentiviral pathogenesis and for AIDS vaccine development.

In the presence of rhesus tetherin, deletion of the SIV *nef* gene greatly impaired virus release compared to wild-type SIV. Expression of the SIV Nef protein *in trans* rescued virus release from cells expressing rhesus tetherin, thereby suggesting a role for Nef in overcoming this restriction in SIV-infected macaques. However, SIV Nef did not enhance virus release in the presence of human tetherin. In the case of HIV-1 Vpu, the situation was reversed. HIV-1 Vpu facilitated virus release in the presence of human tetherin, but not in the presence of rhesus tetherin. Thus, the activities of both HIV-1 Vpu and SIV Nef in counteracting restriction by tetherin are species-specific. These results suggest that, similar to TRIM5α and APOBEC3G [40],[41],[42],[43],[48],[49],[50], species-specific differences in tetherin contribute to the host-range specificity of HIV-1 and SIV.

Deletion of the SIV *env* gene also impaired virus release from cells transfected with proviral DNA. Compared to the deletion of *nef*, this effect was lower and did not appear to be specific for either human or rhesus tetherin. Since the envelope glycoproteins of certain HIV-2 isolates, such as ROD10 and ST, have Vpu-like activity that can enhance the release of diverse retroviruses from otherwise restrictive cell types [28],[29],[30],[31], and the Ebola glycoprotein was recently shown to counteract tetherin [26], this raised the possibility that the envelope glycoprotein of SIV might also antagonize restriction by tetherin. However, under conditions of envelope overexpression, in which the HIV-2 ROD Env could partially rescue particle release, we did not observe a significant effect of SIV Env on virus release. While these results do not preclude a minor contribution of the SIV envelope glycoprotein, they suggest that Nef is the predominant viral gene product by which SIV counteracts restriction by tetherin.

The specificity of SIV Nef for rhesus tetherin mapped to a four amino acid sequence, G_14_DIW_17_, in the cytoplasmic domain of the molecule. These residues closely coincide with a five amino acid sequence, G_14_DIWK_18_, that is missing from human tetherin [64]. Comparison of the predicted amino acid sequences of human versus chimpanzee tetherin revealed that a similar five amino acid sequence (D_14_DIWK_18_) was retained in the cytoplasmic domain of the chimpanzee orthologue [65]. This suggests that a recent 15-nucleotide deletion, that occurred since the divergence of humans and chimpanzees from a common ancestor approximately 5 million years ago [65], accounts for the inability of Nef to counteract restriction by human tetherin. It is therefore tempting to speculate that our ancestors may have encountered a viral pathogen, perhaps a lentivirus related to SIV, that selected for the loss of these sequences at some point during the course of human evolutionary history.

The residues required for SIV Nef recognition of rhesus tetherin are also present in the cytoplasmic domain of sooty mangabey tetherin, and Nef alleles of primary SIV_smm_ isolates were able to overcome restriction by tetherin orthologues from both of these Old World primate species. Thus, the ability of SIV Nef to oppose tetherin appears to have been conserved upon cross-species transmission of SIV_smm_ from sooty mangabeys to rhesus macaques [32],[34],[36]. Although somewhat less efficiently, Nef alleles of HIV-1 and HIV-2, were also able to rescue virus release in cells expressing sooty mangabey and rhesus macaque tetherin. These observations suggest that conserved sequences in Nef, maintained perhaps as a consequence of their role in other functional activities, may contribute to a basal level of activity against macaque and mangabey tetherin. However, consistent with the absence of sequences in the cytoplasmic domain essential for recognition by Nef, none of the Nef alleles tested, with the possible exception of HIV-1 NA7 Nef, rescued virus release in the presence of human tetherin. Hence, this may explain why this activity appears to have been assumed by the envelope glycoproteins of certain HIV-2 isolates [28],[29],[30],[31].

HIV-1, and other enveloped viruses, assemble and bud from cholesterol-rich lipid rafts at the plasma membrane [15],[16],[17],[18],[19]. Nef localizes to lipid rafts and increases virus budding and infectivity [66],[67]. Since tetherin also associates with lipid rafts by virtue of its GPI anchor [13], the ability of Nef to counteract tetherin may be dependent on its association with cellular membranes and targeting to lipid rafts. Consistent with this possibility, amino acid substitutions in the myristoylation site and a putative cholesterol recognition motif of SIV Nef impaired its ability to counteract rhesus tetherin.

Although the mechanism by which Nef counteracts tetherin remains to be fully defined, we found that SIV Nef could downregulate rhesus tetherin from the cell surface. As with the ability to rescue particle release, this activity was species-specific, since SIV Nef was unable to downregulate human tetherin. Furthermore, mutations in the myristoylation site and cholesterol recognition motif that impaired the ability of Nef to facilitate virus release also disrupted its ability to downmodulate rhesus tetherin. These results suggest that, similar to the previously documented ability of Vpu to dowmodulate human tetherin [2], the downregulation of rhesus tetherin by Nef may contribute to the ability of SIV to overcome this restriction in Old World primates.

Treatment with IFNα upregulated tetherin and impaired the release of *nef*-deleted SIV from an infected rhesus macaque cell line. These results confirmed that the expression of tetherin on rhesus macaque cells is interferon-inducible, and similar to HIV-1 *Δvpu* infection of human cells [11], SIV *Δnef* is particularly sensitive to IFNα. Regulation of tetherin by interferon may explain, at least in part, why deletion of the *nef* gene does not result in a more complete block to SIV replication in primary PBMC. While Nef clearly confers an advantage to SIV replication in primary macaque lymphocytes [55], depending on the culture conditions, SIV *Δnef* can replicate nearly as well as wild-type virus. In the absence of interferon, low basal levels of tetherin on primary CD4^+^ lymphocytes may not impose a stringent barrier to virus replication.

A role for Nef in overcoming restriction by tetherin may, nevertheless, help to explain the attenuated phenotype of *nef*-deleted strains of SIV in rhesus macaques [68],[69]. Peak viral loads in animals infected with SIV *Δnef* are typically 1.5 logs lower than peak viral loads in animals infected with wild-type SIV [68]. Greater susceptibility to inhibition by tetherin may contribute to this attenuated phenotype, particularly during the acute phase of infection prior to the onset of adaptive immunity. However, the loss of a number of other functional activities of Nef may also play a role, including CD4 downregulation [70], selective MHC class I downregulation [71], cellular activation [72], and infectivity enhancement [59]. Additional studies to differentiate the role of Nef in opposing tetherin from its other functional activities will be necessary to ascertain the relative importance of the loss of this activity to the attenuation of SIV *Δnef*.

Infection of macaques with simian-human immunodeficiency virus (SHIV) strains in which the *nef* sequences of SIV_mac_239 were replaced by *nef* alleles of HIV-1 demonstrated that HIV-1 Nef can, at least in part, substitute for SIV Nef to facilitate virus replication *in vivo*. Of 19 animals infected with these SHIVnef strains, 11 maintained moderate to high viral loads and 8 progressed to AIDS within 1–2 years of infection [73]. HIV-1 *nef* sequences were retained in all animals and sequence changes predicted to optimize translation were observed suggesting that the expression of HIV-1 Nef conferred an advantage to virus replication [73]. Nevertheless, while the viral loads in these animals were generally higher than in animals infected with SIV *Δnef*, they were not as high, or as consistent, as viral loads typically observed in animals infected with wild-type SIV_mac_239 [73]. This intermediate phenotype is consistent with our present observations indicating that HIV-1 Nef is poorly adapted for counteracting viral inhibition by rhesus tetherin. Although Nef alleles of HIV-1 were less effective than Nef alleles of SIV, HIV-1 Nef appears to have a basal level of activity against rhesus tetherin. Partial activity of HIV-1 Nef against rhesus tetherin, together with other conserved activities of the protein, such as cellular activation and infectivity enhancement [59],[72], may account for the replicative advantage of SHIVnef relative to SIV *Δnef*. However, incomplete adaptation to rhesus tetherin may also have contributed to the somewhat more attenuated phenotype of these viruses compared to wild-type SIV.

Similar to previous reports [1],[2],[52], we observed a greater effect of Vpu when HIV-1 release was measured by an infectivity assay than by an assay for viral antigen. In the presence of human tetherin, expression of the Vpu protein *in trans* resulted in a 27.1-fold increase in the amount of infectious virus versus a 3.7-fold increase in the amount of p24 for a *vpu*-deficient strain of HIV-1. Unlike the infectivity enhancement afforded by Nef, this effect was dependent on human tetherin and was only observed for HIV-1. Hence, this activity appears to be directly related to the role of Vpu in overcoming restriction by tetherin. This suggests that, in addition to facilitating the detachment of virus particles from infected cells, Vpu may also play a role in infectivity enhancement. One possibility is it that Vpu-mediated downregulation and degradation of tetherin may prevent the incorporation of tetherin into virions and reduce the formation of viral aggregates, as suggested by some electron micrographs [1],[11]. This would effectively increase the titer of infectious virus, since more particles would be available to infect a greater number of cells. Alternatively, the observed increase in infectivity may be related to the recently reported effect of Vpu on preventing the accumulation of Env in late endosomes [74]. By inhibiting Env transport to endosomes, Vpu may facilitate the incorporation of Env into virions assembling at the plasma membrane, thereby increasing particle infectivity.

Vpu has a transmembrane anchor at its N-terminus, and mutations within this region disrupt its ability to facilitate virion release [61],[62],[63]. Van Damme *et al.* further demonstrated that the correct amino acid sequence of the Vpu transmembrane domain is required for downmodulation of tetherin from the cell surface [2]. By exchanging the membrane-spanning domains of human and rhesus tetherin, we mapped the sequences responsible for Vpu recognition to the transmembrane domain of human tetherin. This finding was independently verified by McNatt *et al.* in a recent study showing that a combination of amino acid differences within the transmembrane domain, rather a single amino acid subsitution, accounted for differences in the susceptibility of human and rhesus tetherin to Vpu [51]. These results suggest that the mechanism by which Vpu antagonizes tetherin likely requires specific interactions, either direct or via a bridging factor, between the transmembrane domains of Vpu and tetherin.

The inability of Vpu to counteract restriction by rhesus tetherin represents a potential additional barrier to HIV-1 replication in macaques. Efforts to overcome the known species-specific blocks to HIV-1 infection of macaques imposed by TRIM5α and APOBEC3G have led to the construction of recombinant HIV-1 strains containing SIV capsid and Vif sequences [75],[76]. These chimeric viruses replicate efficiently in PBMCs from rhesus and pig-tailed macaques [75],[76], but are unable to sustain persistent, high-level virus replication in animals [77],[78]. Unlike APOBEC3G and TRIM5α, tetherin does not represent an absolute block to infection, but may serve to attenuate virus replication in the absence of effective viral countermeasures. Thus, the susceptibility of these HIV-1 recombinants to restriction by tetherin, perhaps under conditions of interferon-induction *in vivo*, may contribute to the inability of these viruses to replicate efficiently in animals. The introduction of additional sequence changes to overcome restriction by tetherin in macaques therefore represents a promising approach for further adapting HIV-1 for replication in non-human primates.

## Materials and Methods

### Plasmid DNA constructs

#### (i) BST 2 expression constructs

Human *BST2* (*hBST2*) was amplified by PCR from a cDNA clone obtained from the Harvard Plasmid Database. Rhesus macaque and sooty mangabey *BST2* (*rBST2*) were amplified by RT-PCR from mRNA isolated from lymphocytes. *BST2* sequences were cloned into the *Kpn* I and *Xho* I sites of pcDNA3 (Invitrogen, Carlsbad, CA). Amino acid substitutions were introduced into rhesus BST2 by QuickChange site-directed mutagenesis (Stratagene, La Jolla, CA) of pcDNA3-rBST2. Recombinants of human and rhesus tetherin were generated by PCR overlap extension [79] using both human and rhesus tetherin as template and cloned into pcDNA3. All plasmid DNA expression constructs were sequence confirmed.

#### (ii) HIV-1 and SIV clones

Full-length clones for SIV_mac_239, SIV_mac_239 Δ*env*, SIV_mac_239 Δ*nef* and SIV_mac_239 Δ*env*Δ*nef* were constructed from previously described deletion mutants based on p239SpSp5′, pSP72-239-3′, pSP72-239-3′Δ*Env* and pSP72-239-3′Δ*nef* [68],[80],[81],[82]. HIV-1 NL4-3 (pNL4-3) was obtained through the AIDS Research and Reference Reagent Program, Division of AIDS, NIAID, NIH from Dr. Malcolm Martin [83]. The *vpu*-deleted clone of HIV-1 NL4-3 (HIV-1 *Δvpu*) was made by deleting nucleotide 16 of *vpu*, which resulted in a frame shift followed by multiple in-frame stop-codons. This clone was provided by Dr. Swee Kee Wong (New England Primate Research Center, Harvard Medical School). The HIV-1 HXB2 clone pHXB2 does not express Vpu as a result of a defective ACG start-codon. This clone also contains an additional frame shift mutation in *vpr* and premature stop-codons in *tat* and *nef* [84],[85].

#### (iii) Nef, Vpu and Env expression constructs

The SIV Nef expression construct pCGCG-239-Nef was provided by Dr. Jacek Skowronski (Cold Spring Harbor Laboratory, Cold Spring Harbor, NY) [86]. Constructs expressing SIV Nef mutants with amino acid substitutions in the N-terminal myristoylation site (G_2_A) and the cholesterol recognition motif (*crm^−^*; L_129_R, Y_133_A and Y_134_A) were created by site directed mutagenesis of pCGCG-239-Nef. Additional constructs expressing Nef alleles of SIV_smm_FYr1, SIV_smm_FWr1, HIV-2 ROD10, HIV-2 ROD14, HIV-2 CBL-23, HIV-2 60415K, HIV-1 NL4-3 and HIV-1 NA7 HIV-1 were generated by introducing PCR-amplified *nef* sequences into the *Xba* I and *Mlu* I sites of pCGCG. The Nef alleles of SIV_smm_FYr1, SIV_smm_FWr1, HIV-2 CBL-23, HIV-2 60415K, HIV-1 NL4-3 and HIV-1 NA7 were amplified from HIV-1 NL4-3 based expression constructs provided by Dr. Frank Kirchhoff (Institute of Virology, Universitätsklinikum, Ulm, Germany) [87], and the Nef alleles of HIV-2 ROD10 and HIV-2 ROD14 were amplified from pROD10 and pROD14 provided by Dr. Klaus Strebel (NIAID, Bethesda, MD) [28],[30]. The codon-optimized Vpu expression construct pcDNA-Vphu was obtained through the AIDS Research and Reference Reagent Program from Dr. Stephan Bour and Dr. Klaus Strebel [88]. The codon-optimized SIV Env expression construct 64S was obtained from Dr. George Pavlakis (NCI-Frederick, Frederick, MD) [89]. The expression constructs pSA91-HIV-2 ROD10 and pSA91-HIV-2 ROD14 for the HIV-2 ROD10 and ROD14 envelope glycoproteins were provided by Dr. Paula Cannon (Childrens Hospital, Los Angeles, CA) [31].

### Virus release assays

To assay for virus restriction, 293T cells were co-transfected with proviral DNA clones based on HIV-1 NL4-3 (20 ng) and SIV_mac_239 (100 ng) together with pcDNA3-based expression constructs for either human or rhesus tetherin (0–200 ng of pcDNA3-hBST2 or pcDNA3-rBST2). Differences in the amount of plasmid DNA in each transfection were offset by the addition of the empty pcDNA3 vector (0–200 ng). To assay for the ability of Vpu and Nef to rescue virus release in the presence of tetherin, 293T cells were co-transfected with; (1) proviral DNA for either SIV_mac_239 *Δnef* or HIV-1 HXB2 (100 ng), (2) pcDNA3-based expression constructs for human tetherin (pcDNA3-hBST2), rhesus macaque tetherin (pcDNA3-rBST2), sooty mangabey tetherin (pcDNA3-sBST2) or an empty vector control (pcDNA3) (50 ng), and (3) expression constructs for Vpu (pcDNA-Vphu), Nef (pCGCG-239-Nef and derivatives) or vector alone (pCGCG) (100 ng). Transfections were performed in duplicate in 24-well plates seeded the day before at 5×10^4^ cells per well using GenJet Lipid Transfection Reagents (SignaGen Laboratories, Gaithersburg, MD). Forty-eight hours after transfection, the amount of virus released into the cell culture supernatant was measured by SIV p27 and HIV-1 p24 antigen-capture ELISA (Advanced Bioscience Laboratories, Inc., Kensington, MD). The percentage of maximal particle release was calculated by dividing the mean p24/p27 release in the presence of tetherin by the mean p24/p27 release in the absence of tetherin and multiplying by 100.

### Infectivity assays

The amount of infectious virus present in the cell culture supernatant was measured by infection of GHOST X4/R5 cells [50]. These cells harbor a Tat-inducible GFP reporter gene and are susceptible to both HIV-1 and SIV infection by virtue of expressing CD4, CXCR4 and CCR5. The GHOST (3) X4/R5 cells were obtained through the AIDS Research and Reference Reagent Program from Dr. Vineet N. KewalRamani and Dr. Dan R. Littman [53]. These cells were seeded at 2.5×10^4^ cells per well in 24-well plates, and infected the following day by replacing the medium with 100 µl of culture supernatant from 293T cells transfected with HIV-1 or SIV proviral DNA. After 2 hours, the culture volume was brought to 2 ml with fresh medium. Forty-eight hours later, the cells were trypsinized, fixed in 2% paraformaldehyde/PBS and analyzed by flow cytometry. The data were collected using a FACSCalibur flow cytometer (Becton Dickenson, San Jose, CA) and analyzed using FlowJo 8.7 software (TreeStar, San Carlos, CA).

### Western blots

Virions were recovered from cell culture supernatants at 13,000 rpm for 2 hours at 4°C and virus pellets were resuspended in 2× sodium dodecyl sulfate (SDS) sample buffer. Cell lysates were prepared by harvesting cells in 2× SDS sample buffer. Samples were boiled for 5 minutes, separated by electrophoresis on 10% SDS-polyacrylamide gels and transferred to polyvinylidene fluoride (PVDF) membranes using a Trans-Blot SD semidry transfer cell (Bio-Rad, Hercules, CA). The membranes were then blocked in 5% non-fat milk-PBS containing 0.05% Tween-20 for 1 hour and probed overnight with the following primary antibodies at 4°C. SIV p27 and p55 were detected using a cross-reactive monoclonal antibody to HIV-1 p24 (183-H12-5C) at a dilution of 1∶1000. Human and rhesus tetherin were detected using the monoclonal antibody HM1.24, generously provided by Chugai Pharmaceutical Co. (Kanagawa, Japan), at a dilution of 1∶2000. SIV Nef was detected using the 17.2 monoclonal antibody at a dilution of 1∶1000. SIV Env was detected using the KK42 monoclonal antibody at a 1∶1000 dilution. HIV-2 Env was detected using rabbit antisera to HIV-2 ST gp120 at a 1∶1000 dilution. HIV-1 Nef and Vpu were detected using polyclonal rabbit sera at dilutions of 1∶500. β-actin was detected using the monoclonal antibody C4 (Chemicon, Billerica, MA) at a dilution of 1∶1000. The antibodies to SIV Nef, SIV Env, HIV-2 Env, HIV-1 p24, HIV-1 Nef and HIV-1 Vpu were all obtained through the AIDS Research and Reference Reagent Program, NIAID, NIH. Pooled plasma from SIV-infected sooty mangabeys, provided by Dr. Amitinder Kaur (New England Primate Research Center, Harvard Medical School), was used at dilution of 1∶200 for the detection of SIV_smm_ Nef. Pooled plasma from HIV-2-infected individuals, provided by Dr. Walid Heneine (Centers for Disease Control and Prevention, Atlanta, GA), was used at a dilution of 1∶200 for the detection of HIV-2 Nef. After washing in PBS 0.05% Tween-20, blots were probed with HRP-conjugated secondary antibodies, either goat anti-mouse IgG (Pierce, Rockford, IL) or goat anti-rabbit IgG (Sigma), at dilutions of 1∶2000 for 1 hour. Blots were then washed, developed with SuperSignal West Femto Maximum Sensitivity substrate (Pierce), and bands were visualized using a Fujifilm Image Reader LAS 3000 phosphoimager (Fujifilm Photo Film Co., Japan). The pixel intensities for the p27 and p55 bands in virions and cell lysates respectively were determined using the Image J Software (Rasband, W.S., Image, U.S. NIH, Bethesda, MD, http://rsb.info.nih.gov/ij, 1997–2008) as previously described [27].

### Downregulation assay

Stable 293T cell lines expressing HA-tagged human and rhesus tetherin were established by retroviral transduction. An oligonucleotide encoding the HA tag was inserted in-frame into the *Mlu* I sites of *rBST2* and *hBST2* resulting in the introduction of the 9 amino acid HA epitope after residue 131 of hBST2 and after residue 134 rBST2. These HA-tagged *BST2* sequences were then subcloned into the *Age* I and *Pac* I sites of the retroviral vector pQCXIP (Clonetech Laboratories, Mountainview, CA). VSV G-pseudotyped MLV particles packaging these vectors were produced by co-transfection of GP2-293 cells with pVSV-G and used to infect 293T cells. Stable cell lines were selected in medium containing puromycin (4 µg/ml) and enriched for BST2 expression by FACS after staining with the PE-conjugated, anti-HA monoclonal antibody HA.11 (Covance, Emeryville, CA).

Cells expressing HA-tagged human and rhesus tetherin were plated at a density of 5×10^4^ cells per well in 24 well plates and transfected the following day with pCGCG-based constructs (200 ng) that co-express Nef and GFP. After 24 hours, the cells were trypsinized and stained with the anti-HA monoclonal antibody HA.11 for 30 minutes at 4°C. The cells were then washed and stained with an APC-conjugated, donkey anti-mouse IgG polyclonal antibody (eBioscience, San Diego, CA) for 30 minutes at 4°C. The cells were washed, fixed in 2% paraformaldehyde PBS and analyzed using a FACSCalibur flow cytometer (Becton Dickenson, San Jose, CA). The data were analyzed using FlowJo 8.7 software (TreesStar, San Carlos, CA).

### Infection of interferon-treated sMAGI cells with VSV G-pseudotyped SIV *Δenv* and SIV *ΔenvΔnef*

The rhesus macaque sMAGI cell line was obtained through the AIDS Research and Reference Reagent Program from Dr. Julie Overbaugh [90]. Cells were seeded to 24 well plates at a density of 1×10^5^ cells per well in medium lacking or containing 1000 U/ml IFNα (Sigma, St. Louis, MO). The following day, duplicate wells were infected with 50 ng p27 eq. of VSV G-pseudotyped SIV *Δenv* and SIV *ΔenvΔnef*. The medium was replaced after 24 hours continuing with or without IFNα. On day 3 post-infection, the accumulation of p27 in the cell culture supernatant was measured by antigen capture ELISA. The upregulation of tetherin in response to IFNα was assessed by staining sMAGI cells with a FITC-conjugated monoclonal antibody to tetherin (Chugai Pharmaceutical Co.).

### Genbank accession numbers

Human tetherin [D28137]; rhesus macaque tetherin [FJ868941]; sooty mangabey tetherin [FJ864713]; SIV_mac_239 Nef [M33262]; SIV_smm_FYr1 Nef [DQ092760]; SIV_smm_FWr1 Nef [DQ092758]; HIV-2 CBL-23 Nef [DQ222472]; HIV-2 60415K Nef [DQ092764]; HIV-1 NL4-3 Nef [M19921]; HIV-1 NA7 Nef [DQ242535].
